# Complementary Roles of Orexin and Melanin-Concentrating Hormone in Feeding Behavior

**DOI:** 10.1155/2013/983964

**Published:** 2013-07-11

**Authors:** Jessica R. Barson, Irene Morganstern, Sarah F. Leibowitz

**Affiliations:** Laboratory of Behavioral Neurobiology, The Rockefeller University, 1230 York Avenue, New York, NY 10065, USA

## Abstract

Transcribed within the lateral hypothalamus, the neuropeptides orexin/hypocretin (OX) and melanin-concentrating hormone (MCH) both promote palatable food intake and are stimulated by palatable food. While these two neuropeptides share this similar positive relationship with food, recent evidence suggests that this occurs through different albeit complementary effects on behavior, with OX promoting food seeking and motivation for palatable food and MCH functioning during ongoing food intake, reinforcing the consumption of calorically dense foods. Further differences are evident in their effects on physiological processes, which are largely opposite in nature. For example, activation of OX receptors, which is neuronally excitatory, promotes waking, increases energy expenditure, and enhances limbic dopamine levels and reward. In contrast, activation of MCH receptors, which is neuronally inhibitory, promotes paradoxical sleep, enhances energy conservation, reduces limbic dopamine, and increases depressive behavior. This review describes these different effects of the neuropeptides, developing the hypothesis that they stimulate the consumption of palatable food through excessive seeking in the case of OX and through excessive energy conservation in the case of MCH. It proposes that OX initiates food intake and subsequently stimulates MCH which then acts to prolong the consumption of palatable, energy-dense food.

## 1. Introduction

The hypothalamus has long been known to play an important role in feeding behavior. As far back as 1951, Anand and Brobeck [1] reported that bilateral destruction of the lateral hypothalamus (LH) in rats resulted in the complete absence of eating, leading them to term this area of the brain the “feeding center.” Shortly thereafter, Delgado and Anand [2] reported that electrical stimulation of the LH in cats resulted in a 1,000 percent increase in total food intake. Interestingly, rats will work to receive electrical stimulation of the LH (“self-stimulation”), indicating that this nucleus also plays a function in reward, but excessive food intake leads them to decrease their rate of self-stimulation by half [3]. While a number of classical neurotransmitters have been implicated in LH-induced feeding, the discovery of neuropeptides in the brain [4] has led researchers to consider several of these local neuromodulatory neurochemicals as major players in feeding and reward.

Two neuropeptides transcribed in the LH are now understood to play significant roles in feeding and reward. The peptides, orexin A (OX-A) and orexin B (OX-B) (also called hypocretin 1 and hypocretin 2), are cleaved from the 130-amino acid precursor neuropeptide preproorexin (ppOX), which was independently isolated by two research groups in 1998 [5, 6]. Neurons containing orexin (OX) mRNA (about 6700 in the rat) [7] lie exclusively within the hypothalamus, spanning the dorsomedial hypothalamic nucleus through the perifornical area and into the lateral hypothalamic area [5, 6]. This peptide was immediately recognized for its ability to stimulate food consumption, leading one research group to name the peptide OX after the Greek word for appetite, *orexis* [6]. The peptide melanin-concentrating hormone (MCH), isolated from the salmon pituitary in 1983 as an antagonist of alpha-MSH-induced skin darkening [8], was recognized for its role in feeding in 1996 [9]. Neurons containing mRNA for the 165-amino acid precursor prepro-melanin-concentrating hormone (ppMCH, numbering about 12300 in the rat) [7] are distinct from but adjacent to those containing OX, lying predominantly in the LH but also in the perifornical area and subzona incerta [10].

It is now well-established that OX and MCH can act as orexigenic neuropeptides, affecting both food intake and processes of reward that influence food intake. Despite their similar relationship with consumption, these peptides appear to act in complementary rather than redundant ways with food intake, and in fact have largely opposite roles in physiological processes and reward-related behavior. Here, we review the current knowledge about the relationship of these peptides with feeding, while providing a brief discussion of their other actions that may elucidate the mechanisms through which they promote food intake.

## 2. Receptor Function

### 2.1. Intracellular Effects of Receptor Binding 

As with all neuropeptides, the receptors for OX and MCH are G protein-coupled receptors. There are two known receptors for OX, called the orexin 1 receptor (OX1R) and orexin 2 receptor (OX2R), or the hypocretin 1 and 2 receptors. While OX1R binds to OX-A with an affinity that is two to three orders of magnitude greater than for OX-B, OX2R binds to OX-A and OX-B with nearly equal affinity [6]. Orexin receptor binding largely results in neuronal excitation, a rise in cytoplasmic calcium, with OX1R activating G_q_ subunits and OX2R activating G_q_ but also G_i/o_ subunits [6, 11].

Depending on the species studied, there are either one or two receptors for MCH. Rats, mice, hamsters, guinea pigs, and rabbits have only one identified MCH receptor, MCHR1, but humans, rhesus monkeys, dogs, and ferrets also have a functional MCH receptor 2 [12]. As with OX2R, MCHR1 binding appears to activate both G_i/o_ and G_q_ subunits [13], although the major effect of MCHR1 binding is a decrease in cyclic AMP levels [14, 15]. Thus, OX and MCH receptor binding has largely opposite effects on neuronal excitation.

### 2.2. Projections and Receptor Localization 

Projections from OX- and MCH-containing neurons terminate in many of the same brain areas, which may explain why these neuropeptides affect a number of the same behaviors. These brain areas include the locus coeruleus, hippocampus, thalamus, nucleus accumbens (NAc), ventral tegmental area (VTA), amygdala, cortex, and various nuclei of the hypothalamus [16, 17]. The receptors for OX and MCH are also located in these same brain areas [18, 19]. Interestingly, while OX1R and OX2R are often found in the same nuclei, they tend to predominate in different subregions of those nuclei. For example, in the hypothalamus, OX1R is most dense in the anterior hypothalamic nucleus while sparse in the LH and absent from the arcuate and paraventricular nuclei, and OX2R is sparse in the anterior hypothalamic nucleus while dense in the LH, arcuate, and paraventricular nuclei [18].

### 2.3. Interaction between Orexin and MCH 

In support of the idea that OX and MCH work in a complementary or even opposite manner, these two peptides have been shown to interact directly with each other. Neurons containing OX-A or MCH are found to contact each other [20], and OX1R has been described on MCH neurons of the LH [21]. In slice preparation, the addition of OX-A or OX-B evokes long-lasting membrane depolarization and increases spike frequency of MCH cells [22], and the addition of MCH inhibits OX-A-induced spike frequency of OX neurons [23]. Therefore, while OX directly excites MCH neurons, MCH prevents excitation of OX neurons.

## 3. Physiological Effects

The OX and MCH systems largely play opposing roles in the regulation of the sleep-wake cycle and energy balance. Whereas OX promotes wakefulness and energy expenditure and is inhibited by a rise in glucose levels, MCH plays a role in sleep and energy conservation while being activated by glucose. 

### 3.1. Role in Sleep-Wake Cycle 

The firing of OX neurons is robustly tied to arousal during the sleep-wake cycle. These neurons discharge during wakefulness, cease firing with sleep onset, remain silent during slow-wave sleep, discharge periodically during paradoxical (or rapid eye movement, REM) sleep, and begin firing again prior to the transition from REM sleep to waking [24, 25]. They can fire during sleep when an arousing sound stimulus is presented [25, 26]. In support of a direct role for OX in promoting arousal, injection of OX-A or OX-B into the lateral ventricles increases waking and decreases slow-wave and REM sleep [27], while peripheral injection of the OX2R antagonist JNJ-10397049 but not OX1R antagonist SB-408124 decreases the latency for persistent sleep [28]. Transgenic mice lacking the ppOX gene display behaviors strongly resembling narcolepsy, exhibiting frequent periods of behavioral arrest during the dark (active) but not light phase [29]. In fact, the link between OX and narcolepsy was established soon after the neuropeptide's discovery, with an autosomal recessive mutation of OX2R identified in canine narcolepsy [30] and human narcoleptics found to have a reduction, as much as 95%, in the number of OX neurons [31]. Thus OX, acting through OX2R, functions to consolidate the waking state.

In contrast to OX, MCH has been linked with sleep, particularly with paradoxical sleep. Rather than discharging during wakefulness, MCH neurons fire maximally during REM sleep and occasionally during slow-wave sleep [32]. In support of a direct role for MCH in promoting sleep, injection of MCH into the lateral ventricles increases the quantity of paradoxical and slow-wave sleep [33], while both MCH and MCHR1 knockout mice show increased wakefulness [34, 35]. On the other hand, MCH does not appear to play a role in narcolepsy. Human narcoleptics show normal numbers of MCH neurons along with reduced numbers of OX neurons [31, 36], and there is no evidence to date linking mutations of MCHR1 to narcolepsy.

### 3.2. Role in Energy Balance 

Orexin and MCH also largely play opposite roles in energy balance, in parallel with their roles in physiological arousal. For example, intracerebroventricular (ICV) injection of OX potently increases oxygen consumption, and transgenic mice overexpressing ppOX are resistant to high-fat diet-induced obesity due to their increased energy expenditure and reduced fat consumption [37]. A specific role for OX2R in promoting energy expenditure is supported by evidence that this resistance to dietary obesity is found in OX overexpressors lacking OX1R but not in those lacking OX2R and that chronic ICV injection of [Ala11, D-Leu15] OX-B, which binds to OX2R, prevents the development of fat-induced obesity [37]. In contrast to OX, MCH promotes energy conservation. In addition to the small decrease in oxygen consumption that occurs with ICV injection of MCH [38, 39], mice overexpressing the ppMCH gene show increased body weight on a standard diet [40], while those lacking MCH show both decreased body weight and food intake [41, 42]. Interestingly, despite hyperphagia, mice lacking MCHR1 also exhibit decreased body weight on standard chow and are less susceptible to high-fat diet-induced obesity, likely as a consequence of their hyperactivity [43]. Also, genetically obese ob/ob and db/db mice are reported to have elevated MCH mRNA and peptide levels [44], and chronic ICV MCH increases caloric efficiency and body fat mass while an MCHR1 antagonist decreases them [45]. Together, these results support the idea that OX promotes energy expenditure, while MCH reduces it.

### 3.3. Regulation by Glucose 

The activity of OX and MCH neurons is also regulated by energy status as indicated by levels of glucose. A physiological rise in glucose, which would occur after normal meal ingestion, is found to inhibit the electrical excitability of OX neurons in the mouse LH [46, 47]. This is in contrast to nearby MCH neurons, which are excited by elevated glucose levels [46, 48]. Whereas these two changes together might reflect a role for these peptides in energy balance, the evidence described below suggests that they may similarly be seen as promoting intake of a currently consumed food.

## 4. Role in Food Intake

Despite their discordant roles in behavioral state and energy balance, OX and MCH both act as orexigenic neuropeptides. While this effect can be seen with standard laboratory chow, it is even stronger with palatable food. A notable feature of palatable food is that it is generally overconsumed, such that homeostatic signals are overridden during the course of a meal, resulting in prolonged and excessive intake. Importantly, in addition to driving intake, both OX and MCH are themselves stimulated by the consumption of palatable food, further contributing to its overconsumption. While similar in this positive feedback circuit, the stimulation of food intake induced by OX and MCH appears to occur through different, complementary mechanisms. As described later, OX may increase the seeking and motivation to consume palatable food, whereas MCH appears to increase the reinforcing effects of caloric intake.

### 4.1. Effects of Peptides on Food Intake 

A large body of evidence linking OX and MCH with food intake comes from studies of transgenic mice overexpressing or lacking the genes for these neuropeptides and also from studies of outbred rats using injections of the peptides or their antagonists.

#### 4.1.1. Orexin 

As described in the Introduction, the orexigenic effect of OX was noted at the same time that this peptide was first described [6], although the magnitude of its effect is much lower than that of the highly orexigenic neuropeptide Y (NPY) [49]. Under certain paradigms, OX can promote intake of standard laboratory chow in rats and mice. This has been shown for central injection of OX-A into the lateral ventricles [50], hypothalamic paraventricular nucleus [49, 51], LH or perifornical area [51, 52], as well as the NAc shell [53, 54]. While the feeding effects of ICV injection with OX-B are sometimes as potent as those of OX-A [50, 52], these effects of OX appear to occur primarily through OX1R, as a stimulatory effect on food intake with injections into specific brain sites has yet to be observed with OX-B [51, 52]. Notably, ppOX knockout mice show no difference in chow intake when compared to their wild-type littermates [55, 56], supporting the idea that this peptide may not be necessary for normal food intake. 

A body of evidence indicates that the orexigenic effects of OX are far more robust with palatable food. In a variety of paradigms, peripheral administration of the OX1R antagonist SB-334867 is found to significantly suppress intake of palatable, high-fat food [57–59], and injection of OX-A into the third cerebral ventricle selectively increases intake of a high-fat diet more than a high-carbohydrate diet [60]. Whereas peripheral SB-334867 administration does not consistently decrease sucrose self-administration in rats [61–63], *ad libitum* fed ppOX knockout mice consume less of a sucrose solution than their wild-type littermates [64]. This link between OX and palatable food intake, similar to chow intake, appears to be mediated by OX1R, with the OX1R antagonist SB-649868 but not OX2R antagonist JNJ-10397049 found to decrease binge eating of a high-fat, high-sucrose food in female rats [65].

The ability of OX to increase food intake may occur in large part through its stimulation of arousal as well as an increase in motivation, particularly when palatable food is involved. While similar to wild-type littermates in their chow intake, ppOX knockout mice exhibit deficits in their ability to learn about the availability of food. Under mild food restriction, they demonstrate delayed acquisition of operant responding for chow [56] and significantly diminished food-anticipatory activity prior to scheduled feeding [55, 66]. Interestingly, conditional OX gene knockdown via RNAi in normal mice causes decreased responding for chow under both variable and progressive ratio schedules [56], suggesting that OX normally promotes reinforcement-related aspects of food intake. The particular reinforcement-related aspect may be motivation, as OX1R binding largely appears to affect palatable food intake by increasing the motivation for food. Peripheral administration of the OX1R antagonist SB-334867 decreases progressive and fixed ratio responding for a high-fat diet and for sucrose [57, 61, 62], while third ventricle injection of OX-A increases progressive ratio responding for sucrose [57]. Similarly, SB-334867 significantly decreases cue-induced reinstatement of sucrose seeking [61]. Together, these results suggest that OX, acting at OX1R, promotes food intake by increasing the motivation for food reward.

#### 4.1.2. MCH 

The orexigenic effect of MCH on standard chow is roughly of the same magnitude as OX [49]. Intake of chow is increased in rats and mice after injection of MCH into the lateral or third ventricles [9, 49, 67], hypothalamic paraventricular nucleus [68], and NAc shell [69], while it is decreased after injection of an MCHR1 antagonist in the NAc shell [70] or periphery [71]. Although adult ppMCH and MCHR1 knockouts compared to wild-type mice actually demonstrate hyperphagia [41, 43, 72], there is evidence that ppMCH knockouts at a young age consume significantly less chow [41, 42], indicating that MCH has some role in controlling intake of standard food.

Similar to OX, the orexigenic effect of MCH is more robust with palatable food, particularly with calorically dense food. ICV injection of MCH promotes the intake of a high- or medium-fat diet, more than a chow diet [45, 73, 74], and ICV or peripheral injection of an MCH antagonist decreases intake of and operant responding for these diets [45, 75, 76]. Further, transgenic ppMCH overexpressors compared to wild-type mice exhibit increased consumption of a high-fat diet [40]. A similar relationship for MCH is seen with sucrose, with ICV MCH stimulating intake of sucrose solutions [77–79] and systemic administration of the MCHR1 antagonist GW803430 decreasing sucrose self-administration [80]. Notably, the relationship of MCH with palatable food does not extend to sweet, noncaloric saccharin [79, 80], indicating that MCH may be related more to energy conservation than to the intake of palatable food *per se*.

The ability of MCH to stimulate food intake may be due more to its reinforcement of ongoing intake rather than to an effect on the motivation to eat. This is supported by evidence showing that mice lacking the ppMCH gene show decreased responding for a high-fat diet under both fixed and progressive ratio schedules [81] and that Wistar rats given systemic injection of the MCHR1 antagonist GW803430 show reduced fixed and progressive ratio responding for a sucrose solution [80]. Further, although the same injection was found to suppress cue-induced reinstatement of lever pressing for sucrose [80], MCH blockade does not affect reinstatement for fat. Neither peripheral injection of the MCHR1 antagonist SNAP 94847 in rats nor chronic loss of ppMCH in mice significantly affects either cue- or pellet-induced reinstatement of fat seeking [58, 81]. Together, this evidence indicates that MCH promotes food intake primarily by increasing energy conservation, motivating animals to continue consuming energy-dense foods.

### 4.2. Effects of Food Intake on Peptides 

Another set of studies that tie OX and MCH to food intake examines the effects of various feeding conditions on their levels of mRNA or peptide.

#### 4.2.1. Orexin

Neurons containing OX are activated in anticipation of feeding but also following consumption of food, provided that it is palatable food. Food deprivation upregulates gene expression and protein levels of OX (OX-A and OX-B) and both of its receptors in the hypothalamus [82] as early as twenty-four hours after onset. After a forty-eight hour fast, similar changes have been observed [6, 83], and in female rats, the activity of OX neurons is also upregulated, as indicated by double-labeling of OX with phosphorylated CREB [84]. Given the relationship of OX with glucose (see Section 3.3), these changes after food deprivation may reflect lowered glucose levels; however, they may also reflect changes in arousal, as a single day of food deprivation increases wheel running activity [85] and decreases the total number of sleep episodes [86], effects that occur under conditions of heightened OX activity and are taken to indicate foraging behavior.

In line with the effects of food deprivation, OX neuronal activity and gene expression are also upregulated when animals are expecting to receive valued food. In rats with restricted access, OX mRNA and double-labeling of OX with c-Fos are elevated prior to access to a daily meal of chow, corn oil, or chocolate [57, 87], while OX levels begin to return to baseline within 30 minutes after the start of meal consumption [87]. In sated rats, OX and c-Fos double-labeling is also increased by a tone that signals the availability of palatable food in the form of high-sucrose pellets [88]. Siegel and colleagues [89] demonstrated that the expression of Fos in OX neurons increases in animals working for chow during the light phase but not when working to avoid shock or when receiving unearned rewards. These results suggest that OX neurons are activated in conditions when animals expect to receive specific, often preferred foods. In further support of this idea, c-Fos expression in OX neurons is also increased during extinction of sucrose seeking [61], when animals are motivated to obtain food rewards.

After consuming palatable food, OX levels are similarly elevated. With high-fat compared to low-fat, high-carbohydrate food, OX gene expression, and OX-A peptide levels are elevated after a single meal or up to three weeks on the diet [90–92], with longer periods of exposure leading to compensatory decreases in OX [93]. Interestingly, this fat-induced increase in OX may occur more from saturated than unsaturated fat, as consumption of a lard-based diet leads to higher OX mRNA levels than does that of a fish oil-based diet [94, 95]. Similar to the results with fat, OX gene expression is also elevated following consumption of a high-sugar diet [96]. Together, these findings indicate that OX is activated both when animals are seeking food and also after they have consumed palatable food, which may in part explain why these foods are consumed in excess.

#### 4.2.2. MCH

Unlike neurons containing OX, those containing MCH are not consistently activated in anticipation of feeding, although they are activated following consumption of a palatable, caloric food. Gene expression of MCH is upregulated after twenty-four hours of food deprivation [9, 97], although longer periods of fasting either increase peptide levels or leave them unchanged [97–99]. The failure to observe an increase may be due to the sensitivity of MCH neurons to the sleep-wake cycle. This is indicated by evidence showing MCH peptide levels after forty-eight hours of food deprivation to be increased in rats sacrificed during their resting (light) phase, but not their active (dark) stage [100] when MCH neurons are normally quiescent. Alternatively, MCH may play a less prominent role than OX in food seeking induced by deprivation, particularly as MCH neurons are excited by elevated glucose levels (see Section 3.3). Thus, these changes in MCH in response to food deprivation, unlike with OX, may be related more to changes in caloric efficiency than in circulating glucose. 

In contrast to OX, there is little evidence linking MCH with the expectation of receiving food, with double-labeling of c-Fos and MCH in sated rats unchanged by a tone signaling the availability of high-sucrose pellets [88].

Levels of MCH after consuming calorically rich food are clearly increased, supporting its role in palatable food consumption. With maintenance on a high-fat compared to low-fat diet, MCH gene expression and hypothalamic peptide levels as well as hypothalamic MCHR1 mRNA levels are elevated [101, 102]. Although the type of fat in the diet may not make a difference in this effect [95], the caloric content appears to be essential, with drinking of noncaloric saccharin having no effect on MCH gene expression [103]. Thus, consistent with its proposed role described previously in promoting motivation for intake of caloric food, these results indicate that MCH is activated after animals have consumed caloric food, further promoting overconsumption. 

## 5. Interactions with Other Neurochemicals

Whereas OX and MCH each play a significant role in the consumption of palatable food, it is clear that these neuropeptides do not work in isolation. Two classes of neurochemicals with which they directly interact are first-order feeding neuropeptides of the arcuate nucleus and dopamine in the limbic system.

### 5.1. Arcuate Peptides

The orexigenic actions of OX and MCH may be due, in part, to their similar downstream activation of neuropeptides in the hypothalamic arcuate nucleus, NPY and agouti-related protein (AgRP). In the arcuate, OX-positive axon terminals directly contact neurons containing NPY [104], and OX1R protein is located on both NPY and AgRP-containing neurons [21]. Orexin also directly activates NPY neurons, as the addition of OX-A or OX-B to the superfusate of isolated arcuate NPY neurons increases their intracellular calcium content [104]. This translates into effects of OX on feeding, as the orexigenic effect of ICV OX-A or OX-B is greatly reduced by ICV pretreatment with an NPY receptor antagonist [105, 106]. Similarly, chronic ICV injection with MCH, which stimulates feeding, also upregulates NPY gene expression [107], and the orexigenic effect of ICV MCH is significantly diminished by ICV injection of an NPY receptor antagonist [108]. In part then, the similar ability of OX and MCH to promote food intake may be due to their similar downstream effects on other orexigenic neuropeptides.

### 5.2. Limbic Dopamine

The dissimilar motivation and arousal-related actions of OX and MCH may be due more to their opposing downstream effects on the neurotransmitter dopamine (DA), in limbic nuclei such as the NAc and prefrontal cortex (PFC). A rise in OX levels results in DA release into the NAc shell and PFC, which can occur from OX acting directly at DA terminals or at their source in the VTA. Application of OX-A to PFC slices increases phasically evoked DA release, an effect inhibited by the OX1R antagonist SB334867 [109], and application of OX-A or OX-B to VTA slices increases the firing frequency of DA neurons [110]. Similarly, ICV injection of OX-A stimulates c-Fos in VTA DA neurons, primarily in those projecting to the NAc shell and PFC rather than the NAc core [111]. In line with this, ICV OX-A is also found to elevate levels of DA in the PFC but not the NAc core [112]. These findings with OX contrast with those observed with MCH, which through transgenic studies appears to inhibit accumbal DA. Mice lacking ppMCH exhibit increased electrically evoked DA release in the NAc shell and increased DA transporter levels in both the NAc shell and core [81, 113]. As the application of MCH to VTA slices fails to affect firing of DA neurons [110], these DA changes in knockout mice may be due to presynaptic actions in the NAc. These opposite effects of OX and MCH on limbic DA may help to explain their opposite effects on arousal and reward (see Section 6).

It is interesting to consider the similar orexigenic actions of OX and MCH in light of their opposite effects on DA. This neurotransmitter plays an important role in promoting food-seeking behavior [114] and appetitive motivational processes in general [115], but animals will also work to enhance DA levels when they are low [116]. In fact, animals prone to overeating a high-fat diet exhibit markedly reduced basal levels of DA in the NAc [117]. Seemingly then, palatable food intake can be increased from an OX-induced elevation of DA, which increases arousal and seeking of palatable food [118, 119], but it can also be increased by an MCH-induced reduction in DA, which produces anhedonia (see Section 6.2) and the need to restore DA levels through the consumption of palatable food [117]. This suggests the possibility that OX may contribute to the rise in accumbal DA that normally occurs prior to meal consumption, while MCH may contribute to the fall observed during the feeding bout [120].

## 6. Role in Reward

In line with their opposite effects on limbic DA, OX and MCH largely have opposite effects on processes of reward. Whereas OX increases the reinforcing properties of ingested substances, MCH instead appears to promote depression and anxiety.

### 6.1. Orexin

The peptide OX plays a role in reward and reinforcement, acting through OX1R or OX2R. In tests of conditioned place preference (CPP) following pairing with drug administration, VTA injection of OX-A during conditioning induces morphine CPP in a dose-dependent manner [121], and peripheral administration of the OX1R antagonist SB-334867 or OX2R antagonist TCS-OX2-29 suppresses its acquisition and expression [122, 123]. While morphine CPP may be perpetuated by OX binding at OX1R or OX2R, ethanol CPP appears to be mediated more by OX2R. Acquisition, expression, and reinstatement of ethanol CPP are attenuated by peripheral treatment with the OX2R antagonist JNJ-10397049 but not by the OX1R antagonists SB-408124 [124] or SB-334867 [125]. The reinforcing effects of naturally rewarding stimuli may also involve OX. In male rats, conditioned cues associated with sexual behavior in a CPP paradigm induce c-Fos in OX neurons, and lesioning of OX neurons prevents the formation of CPP for a chamber paired with sexual behavior [126]. Thus, OX may be important in reward processing of both drugs of abuse and also natural rewards, such as sexual activity or palatable food intake.

### 6.2. MCH

In contrast to OX, MCH may not promote reward processing but instead is linked with anxiety and depression. Mice with deletions of MCHR1 show no difference from their wild-type counterparts in cocaine- or amphetamine-induced CPP [127] and in fact show hypersensitivity to the locomotor activating effects of the DA psychostimulant d-amphetamine [128], suggesting that these mice have enhanced drug-induced reward processing. Instead, MCH is strongly linked with anhedonia in the forced swim test (FST). The amount of time spent immobile in the FST correlates positively with ppMCH mRNA [129], and MCHR1 antagonists produce an antidepressant effect in the FST when administered peripherally [130] or directly in the NAc shell [70, 131]. Conversely, NAc shell injection of MCH produces the opposite effect [70]. The MCHR1 antagonist SNAP-7941 also acts as an anxiolytic in tests of rat social interaction and guinea pig maternal-separation vocalization [130]. Thus, in contrast to OX, MCH may in some cases act as an antireward neuropeptide.

## 7. Conclusions

In summary, OX and MCH, expressed in the LH and acting through their receptors in many of the same nuclei of the brain, are similar both in promoting the consumption of palatable or caloric food and in being stimulated by the intake of this food, responding in a positive feedback cycle to promote excess consumption. Importantly, they appear to have complementary roles in controlling this feeding behavior, likely through their largely opposite roles in reward systems and motivation, as well as a number of physiological processes, including the sleep-wake cycle, energy expenditure, and glucose metabolism. Perhaps for these reasons, OX and MCH affect different aspects of food intake. The evidence suggests that OX is activated in situations of food seeking and promotes the motivation for food reward, possibly activating the feeding process, whereas MCH plays a larger role during ongoing food intake and reinforces consumption, acting to prolong the intake of energy-dense foods. Thus, for a single meal, OX may be involved in initiating palatable food intake and activating MCH neurons, and after meal initiation and the consequent rise in glucose, MCH functions to prolong the consumption of calories for the sake of energy conservation. These physiological and behavioral effects of OX and MCH can be further understood in light of their downstream effects on other neurochemicals. While these neuropeptides both upregulate NPY which may contribute to their similar orexigenic effects, they have opposite effects on limbic DA, which may contribute to their complementary effects on reward and motivation that themselves can enhance palatable food intake. These two peptides in the LH provide an interesting example of the complex and sometimes opposing physiological, behavioral, and neurochemical processes that are involved in promoting the ingestion of palatable food.
